# Valorization of Residue from Aluminum Industries: A Review

**DOI:** 10.3390/ma17215152

**Published:** 2024-10-23

**Authors:** Andrie Harmaji, Reza Jafari, Guy Simard

**Affiliations:** Department of Applied Sciences, University of Québec in Chicoutimi (UQAC), 555, Boulevard de l’Université, Saguenay, QC G7H 2B1, Canada

**Keywords:** aluminum, valorization, waste heat, dross, salt slag, spent carbon cathode, bauxite residue

## Abstract

Recycling and reusing industrial waste and by-products are topics of great importance across all industries, but they hold particular significance in the metal industry. Aluminum, the most widely used non-ferrous metal globally, generates considerable waste during production, including dross, salt slag, spent carbon cathode and bauxite residue. Extensive research has been conducted to recycle and re-extract the remaining aluminum from these wastes. Given their varied environmental impacts, recycling these materials to maximize residue utilization is crucial. The components of dross, salt slag, and bauxite residue include aluminum and various oxides. Through recycling, alumina can be extracted using processes such as pyrometallurgy and hydrometallurgy, which involve leaching, iron oxide separation, and the production of alumina salt. Initially, the paper will provide a brief introduction to the generation of aluminum residues—namely, dross, salt slag, and bauxite residue—including their environmental impacts, followed by an exploration of their potential applications in sectors such as environmental management, energy, and construction materials.

## 1. Introduction

Aluminum (Al) and aluminum alloys are highly valued metals with diverse applications because of their superior thermal characteristics, electrical conductivity, low weight, and corrosion resistance [1]. As one of the most recycled materials, aluminum’s significant value and extensive use in the construction, automotive, packaging, and aerospace industries highlight its importance. Notably, aluminum used in building and automotive parts is recycled at rates up to 90% at the end of its useful life [2], and 75% of all aluminum ever produced remains in use today [3]. Ranking second only to steel in the amount of non-ferrous metal produced, aluminum’s production volume surpasses that of all other non-ferrous metals combined [4]. In particular, aluminum alloys are crucial in the aerospace and automotive industries, where they often replace steel due to their ability to naturally form a passive oxide layer when exposed to air, thereby preventing corrosion under various conditions [5].

The aluminum industry generates both non-solid residues (e.g., waste heat) and solid waste, including aluminum dross, salt slags, spent carbon cathode, and bauxite residues. These residues from both primary and secondary aluminum production pose a global challenge. The recycling and reuse of industrial waste and by-products are paramount in construction technology. Traditional industrial by-products like fly ash, granulated blast furnace slag, and silica fume have been extensively researched for their reactivity and effectiveness as active additions in cement and concrete manufacturing [6]. Less reactive wastes serve as additional cementitious materials for cement production or as inert aggregates for concrete and mortar. The residues from aluminum industries, rich in metal oxides and containing valuable rare earth elements, underscore the importance of valorizing aluminum production residues for their incorporation and reuse in everyday applications. This review begins with a brief overview of aluminum production and the residues it generates—namely, waste heat, aluminum dross, salt slag, spent carbon cathode, and bauxite residues. We then explore the valorization potential of each residue and present opportunities for large-scale residue reuse.

## 2. Overview of Aluminum

In the following sections, we describe the Hall–Héroult process and explain the technology applied during the production process to fully comprehend the operations of the global aluminum industry. Opportunities for the use of residues can then be determined, and we can identify constraints on integrating novel procedures for minimizing and reusing waste products.

### 2.1. The Hall–Héroult Process

**Figure 1:**
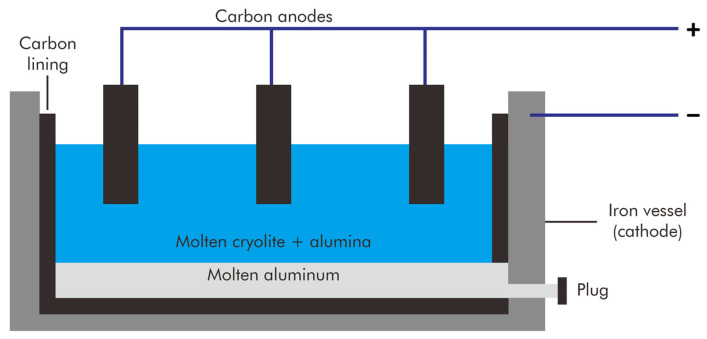
Illustration of the Hall–Héroult process.

The current method of producing primary aluminum was established in 1886 when Paul Héroult and Charles Hall independently discovered that the reduction of molten aluminum oxide in cryolite (Na_3_AlF_6_) served as a more affordable means of aluminum production. As both shared the patent, this production technique is known as the Hall–Héroult process (Figure 1). In 1887, Karl Joseph Bayer discovered that alumina (Al_2_O_3_) could be extracted from bauxite, an ore named after the French province of Les Baux, and used as a cost-effective feedstock for the Hall–Héroult process.

The Bayer process separates alumina from bauxite. Bauxite is dissolved in a hot caustic solution to break it down into a green liquor. This green liquor is then separated from the undissolved material and allowed to cool, where it precipitates pure alumina hydrate. The hydrate is subsequently calcined to produce alumina. The high-purity petroleum coke used to make the carbon anodes for aluminum reduction is ground, calcined, and combined with ground used carbon anodes and pitch, which serves as a binder, to create a paste. Although the Hall–Héroult process consumes a significant amount of electrical energy, manufacturers have gradually increased the efficiency of this technique. Nonetheless, the extraction process produces significant CO_2_ emissions from the anodes and relatively high heat loss from the electrolytic cells.

### 2.2. Current Production of Aluminum

Aluminum is the most abundant metallic element, constituting 7.96% of the Earth’s crust [7]. It is seldom found in its elemental form because of its high reactivity, especially when in contact with oxygen, necessitating its extraction from minerals in ores. Bauxite is the principal ore used for aluminum extraction, accounting for more than 99% of primary aluminum production [8]. Open-cast mines on the Earth’s surface are the main sites for bauxite extraction [9]. Bauxite contains three main types of aluminum hydroxide compounds: gibbsite (Al(OH)_3_), diaspore (α-AlO(OH)), and boehmite (γ-AlO(OH)) [10]. Typically, alumina constitutes 40–60% of bauxite’s weight, along with trace amounts of iron, silicon, and titanium compounds [3], necessitating refinement because of these trace compounds.

Advancements in the metallurgy of aluminum alloys, along with population growth and increased economic activity [11], have led to a rise in bauxite extraction. In 2023, the world’s aluminum production increased marginally to 70 million metric tons (MT) from 68.4 million MT in 2022. Of this total, 41 million metric tons, or more than half, came from China, followed by India and Russia [12]. According to official data, China’s primary aluminum output increased by 7.10% year over year to 14.24 Mt between January and April of this year. An unsustainable increase in alumina imports (+75% y/y to 1.06 Mt), the intermediate product between bauxite and metal in the primary aluminum production chain, is the main cause of this growth [13]. In this country, alumina is produced with an estimated 82 million metric tons produced, followed by Australia with about 19 million metric tons that year [14]. The largest producer of aluminum in India, Vedanta Aluminum, ranks among the top three global companies, with a production capacity of 3 million tonnes per annum (MTPA) [15]. The company announced the new 1.5 MTPA expansion at its alumina refinery in Lanjigarh, Odisha in early 2024. As a result, the alumina refinery’s production capacity has grown from its previous 2 MTPA to 3.5 MTPA. Global demand for aluminum is expected to double over the next decade [16], though more conservative estimates suggest a potential doubling or tripling of demand by 2050 [17]. Australia, China, Guinea, Brazil, India, and Indonesia are the six largest bauxite producers currently [18]. While bauxite ore is plentiful, the growth in secondary production and recycling of aluminum has also surged; hence, the estimated 55–75 Gt of global bauxite reserves are likely to meet demand for many centuries [19].

**Figure 2:**
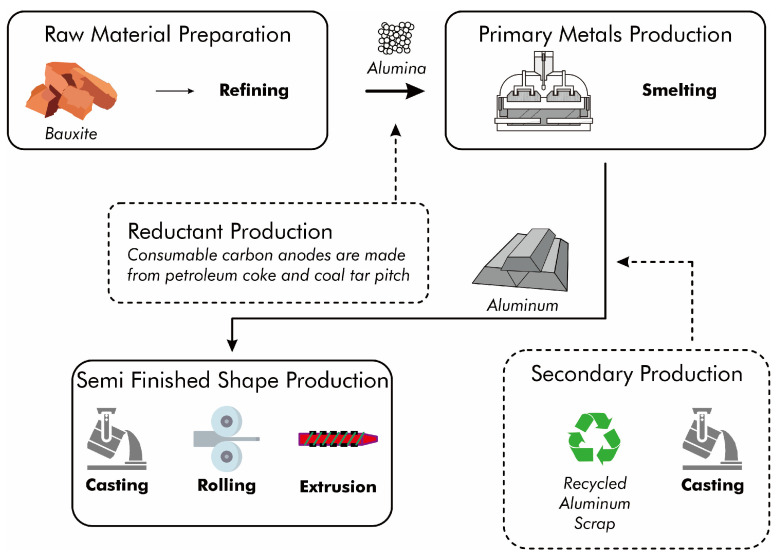
Flow diagram of primary and secondary aluminum processing.

Primary aluminum is produced exclusively from mined ore. The refined ore undergoes electrolytic reduction (Figure 2) before being shaped through casting, rolling, or extrusion to produce bulk material. For products with specific requirements, manufacturers process this bulk material further. Secondary aluminum production involves scrap pretreatment as well as smelting and refining. Pretreatment operations include the processing, cleaning, and sorting of scrap. The smelting and refining processes encompass cleaning, melting, refining, alloying, and pouring of aluminum recovered from scrap.

### 2.3. The Bayer Processes

The Bayer process is the most widely recognized refining method, though the nepheline-based process and the combined or parallel Bayer–Sinter process are used in some regions. The nepheline-based process uses nepheline syenite as an alternative to bauxite [20], prized for its high aluminum content. However, it presents challenges because of (a) the slow dissolution of aluminum-bearing phases and (b) the formation of silica gel at high pulp densities and low treatment temperatures [21,22].

**Figure 3:**
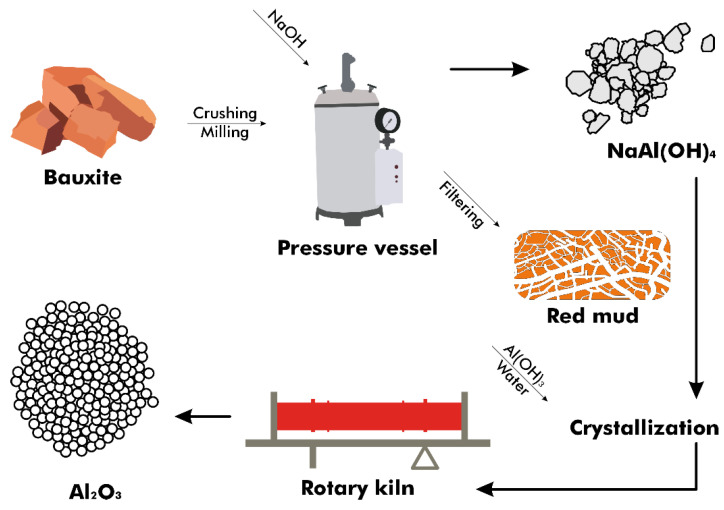
Flow diagram of the Bayer process.

In the Bayer process, bauxite ore undergoes processing to produce purified alumina, necessitated by the impurities within the ore (Figure 3). High-porosity and reactive activated alumina are produced in situ from the bauxite rocks with minimal processing, without direct purification from the ore [23]. This process involves adding ground and blended bauxite and sodium hydroxide (NaOH) to a pressure vessel at temperatures ranging from 150 to 265 °C, depending on the concentration of aluminum compounds present [24]. The compounds dissolve, creating an equilibrium as described by Equations (1) and (2) [25], with conditions favoring the reaction’s shift to the right to produce hydrated NaAl(OH)_4_ in a step known as digestion.

(1) $$Gibbsite:Al{\left(OH\right)}_{3}+{Na}^{+}+{OH}^{-}⇌Al{{\left(OH\right)}_{4}}^{-}+{Na}^{+}.$$

(2) $$Diaspore\;and\;Boehmite:Al{\left(OH\right)}_{3}+{Na}^{+}+{OH}^{-}⇌Al{{\left(OH\right)}_{4}}^{-}+{Na}^{+}.$$

A supersaturated sodium aluminate liquor known as a slurry is produced by chemically treating the solution. The equilibrium is then shifted to the left by reversing Equation (3) and by cooling the mixture in the presence of seeded crystals of Al(OH)_3_. Aluminum hydroxide is heated at approximately 1000 °C in a rotary kiln or calciner to remove water and produce anhydrous aluminum oxide, while the sodium hydroxide is recycled [26].

(3) $$2Al{\left(OH\right)}_{3}\to {Al}_{2}O_{3}+3H_{2}O$$

**Figure 4:**
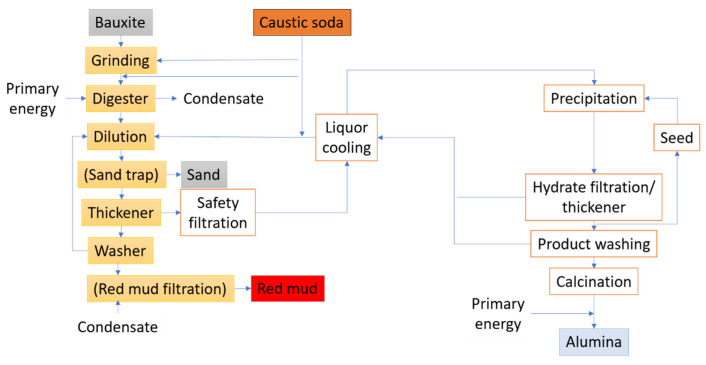
Overview of the stages of refining bauxite to alumina.

The primary smelters or other industries then acquire the anhydrous aluminum oxide for processing. Figure 4 presents the stages of a typical refining procedure.

## 3. Impact of Aluminum Production on the Environment

The manufacturing of aluminum involves numerous additional supporting activities and resources, all of which must be considered in any life cycle analysis of aluminum production. The impacts of aluminum production include the construction of roads and infrastructure, as well as the extraction and disposal of a large amount of rock. Once the mine site is depleted, the post-restoration soils exhibit low water-holding capacities, rendering them unsuitable for crop cultivation. Furthermore, aluminum production is associated with the damage and even loss of ecosystems through photochemical ozone formation, acidification, eutrophication, and ecotoxicity [27]. The aluminum industry emits between 0.45 and 5 Gt of carbon dioxide (CO_2_) equivalent annually [28]. Industrial facilities converting alumina into aluminum often rely on power plants powered by fossil fuels, thereby increasing greenhouse gas emissions. Power generation may also include hydroelectricity, which reduces carbon emissions but still poses environmental impacts related to dam construction and the flooding of large areas for reservoirs. Particulate and gaseous emissions during aluminum reduction processes include carbon monoxide (CO), CO_2_, sulfur oxides (SOx), aluminum fluoride (AlF_3_), calcium fluoride (CaF_2_), and volatile organic compounds. Polycyclic aromatic hydrocarbons (PAHs), pollutants created during the electrolytic process, are particularly concerning given their carcinogenic potential. Hence, any future sustainable plant design must prioritize the efficient use of natural resources, minimize the input of residues into the environment, and explore alternative, recyclable energy sources such as waste heat.

### 3.1. Waste Heat

**Figure 5:**
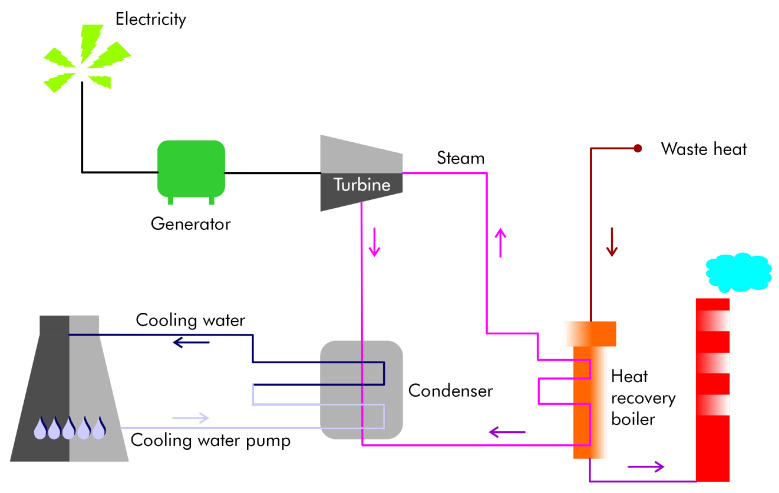
A heat recovery model. The steam is fed into the highly efficient turbine/generator unit, which generates electrical energy.

Primary aluminum production consumes significantly more energy and water than secondary production. The efficiency of existing plants can be improved using modern technologies. Recovering, reclaiming, and using generated waste heat are methods for reducing energy consumption. Heat recovery techniques, including the use of electrolytic cells, can help minimize heat loss and reduce the need for fuel and electricity. Most heat is lost through the cell sidewalls, with a smaller amount lost through off-gassing. In secondary aluminum production, scrap melting in high-temperature furnaces, a crucial step, only employs waste heat recovery in about one-third of existing high-capacity furnaces. Nevertheless, several methods for recovering waste heat are available (Figure 5).

### 3.2. Aluminum Dross

A significant challenge in producing pure aluminum is the generation of other solid wastes. Primary aluminum production generates considerably more atmospheric emissions and solid waste than secondary production. Materials with less than 45% aluminum content are classified as “dross”, whereas residues with more than 45% aluminum content are termed “skimmings” [29]. Dross can be further divided into “white dross”, from primary smelters without salt covers, and “black dross”, originating from secondary smelters. Metal beads, crystallized salt, and solid nonmetallic particles are found in the nonmetallic waste products from dross smelting processes. White dross comprises a fine powder obtained by skimming the molten aluminum, containing between 20% and 45% recoverable metallic aluminum. In the second stage of aluminum production, the flux-containing dross resulting from the melting operation of aluminum scrap in reverberatory furnaces is referred to as “black dross”, distinguished by its darker color [30]. Black dross typically contains aluminum oxide (20–50%), a salt–flux mixture (40–55%), and aluminum metal, with the recoverable aluminum content usually ranging between 10% and 20%. It has a much higher salt content (typically greater than 40%) than white dross [31]. In the separation process of aluminum production, white dross requires more energy and water than black dross and also produces more waste [32]. Globally, more than 95% of the four million tonnes of white dross and over a million tonnes of black dross produced annually are landfilled.

### 3.3. Salt Slag

The nonmetallic residue resulting from dross smelting operations is known as salt slag and typically contains 3–7% residual metallic aluminum, 15–30% aluminum oxide, 30–55% sodium chloride, and 15–30% potassium chloride, along with other materials that vary in abundance depending on the type of scrap [33]. Salt slag, also referred to as salt cake, is classified as toxic and hazardous waste with variable harmful properties [34]. The scrap/dross and salt flux are re-added to an oil or gas-fired furnace during the rotary salt furnace process. The salt facilitates the agglomeration and separation of the metal, thereby increasing metal recovery by shielding it from the atmosphere’s reactivity [35]. Additionally, it enhances heat transfer to the metal, prevents metal oxidation, and absorbs contaminants including oxides, nitrides, carbides, and other substances found in scrap or created by reactions during melting [36]. Aluminum metal and salt slag are removed from the furnace after melting. After tapping and cooling, the salt cake is formed, containing all the nonmetallic components from the raw mix [37]. The oxides in the dross (from the raw mixture) form a net-like structure, trapping aluminum. This structure is disrupted by the molten flux, which also facilitates the coalescence and sinking of aluminum droplets into the aluminum bath [38]. Carbon typically remains in the salt slag following the decomposition of organic contaminants. If insufficient amounts of salt are present, a high concentration of oxides and other contaminants may cause the molten salt to become highly viscous. In practice, a more viscous slag results in significant metal loss because of the entrapment of metal droplets.

Carbides, nitrides, and phosphates bound with aluminum, along with the oxides of alloying elements (e.g., Cu, Fe, Si, Zn), make up most of the nonmetallic compounds. When liquid aluminum comes into contact with finely dispersed carbon, Al_4_C_3_ is formed, which originates from organic contaminants in scrap such as paints, plastic coatings, and hybrid-sandwich components. The AlN-containing dross is fed into rotary furnaces, where it is captured by the salt slag. The high concentration of NaCl and KCl in salt slag causes the slag to release chlorides into water when in contact with a water source or groundwater. The presence of toxic, harmful, explosive, poisonous, and foul-smelling gases, e.g., NH_3_, CH_4_, PH_3_, H_2_, and H_2_S, means that the gaseous emissions resulting from the contact of the salt slag with water may also have a significant negative impact on the environment [39]. Depending on the type of scrap, it may contain carbides, nitrides, phosphides, and sulfides [40].

### 3.4. Spent Carbon Cathode

Another solid waste produced from aluminum is the spent carbon cathode. This carbon-rich material produced when electrolysis cells in the aluminum manufacturing process are overhauled [41]. Most aluminum electrolytic cells only last 5 to 8 years because high-temperature electrolytes continuously erode the cathode carbon during production [42]. The main way that SCC is currently disposed of is in landfills [43], which may release highly toxic cyanide and soluble fluoride into the environment, endangering ecological and environmental safety [44]. Moreover, it also has a significant effect on the wellbeing and ecological equilibrium of plants and animals [45]. While carbon recovery is relatively easy, recovering fluorine resources is typically difficult, primarily for innocuous detoxification [46]. Among the techniques used to treat spent SCC are high-temperature heating [47], chemical leaching [48], and physical separation [49]. The drawbacks of chemical leaching and physical separation are their lengthy processing times, complicated procedures, and high wastewater production. The combustion and boiling points of dangerous compounds, like fluorides, serve as the foundation for the high-temperature heating treatment. Moreover, these techniques have a number of drawbacks, including low leaching rates, lengthy leaching cycles, high energy consumption, low recovery rates, insufficiently pure carbon powder, and the production of new pollutants throughout the treatment process [50]. Therefore, finding a treatment method that is both effective and pollution-free is essential.

### 3.5. Bauxite Residue

The most significant waste created in the primary aluminum process is bauxite residue or “red mud”, generated during the Bayer process. This red-colored sludge is produced in the second stage of alumina production. The high concentration of Fe_2_O_3_ in bauxite residue gives it its red-colored appearance. To produce one ton of aluminum, four tons of bauxite are required, and two tons of bauxite residue are generated. Red mud consists primarily of iron (Fe), silicon (Si), and titanium (Ti) oxides, sodium hydroxide, unextracted aluminum oxide, zinc (Zn), phosphorus (P), nickel (Ni), and vanadium (V), as well as other oxides. The composition of red mud varies among aluminum production facilities (Table 1).

The bauxite residue becomes extremely alkaline, with pH values from 9 to 13, when bauxite is treated with concentrated NaOH at high temperature and pressure [71]. Bauxite residue waste management typically involves controlled landfill disposal. Because sodium hydroxide is added during the Bayer process, the residue is extremely alkaline [72]. In Canada, red mud is considered a Class 8 hazardous good (corrosive material) under the dangerous goods transport regulation, Sections 2.40–2.42, in accordance with UN code No. 3244. Thus, specific packaging and the use of UN-compliant containers are required for all shipments. Transportation via airfreight is quite difficult, except in limited quantities. Meanwhile, sea freight is easier, and road transportation packaging standards are normally used. Moreover, given the high alkalinity of the water and soil (pH 10–13), the presence of heavy metals, and even traces of radioactive elements, there is a significant contamination risk [73]. Extensive treatment involving maintenance and monitoring is necessary, as is a sizable storage space for its disposal.

## 4. Valorization of Residue from Aluminum Production

Given its high pH and trace amounts of heavy metals, aluminum residue poses a serious environmental risk. The pursuit of producing cleaner bauxite residue has become a key focus for the aluminum industry, necessitating that governments and producers adopt a circular economy model for aluminum production, use, and reuse [74]. The circular economy model facilitates waste reduction by adding value to products [75]. Aluminum is one metal that benefits significantly from the circular economy; it can be recycled numerous times without losing its mechanical, physical, or chemical properties. The following subsections discuss strategies to promote the valorization of waste heat, white and black dross, salt slag, and bauxite residue from aluminum manufacturing.

### 4.1. Waste Heat Recovery

Several studies have focused on the use of waste heat from the aluminum industry, which could benefit from potential sources of recoverable heat, such as those from refining, smelting, recycling, and secondary melting processes. Heat recovery is also feasible from calciners and hot alumina. For calciners, various technologies are under consideration to recover latent heat from water vapor [25]. There is potential to use recoverable heat from hot alumina to produce heated water for generating electricity or for other processes within aluminum processing plants. However, the availability of hot water for plant operations is often limited.

Electrolysis pots have shown good potential for heat recovery. The steel-shell-protected wall can be fitted with a thermoelectric device. In Europe, heat exchangers on the exhaust gas stream are used to recover heat from aluminum smelting cells or reactors [26]. Hot exhaust gases from fuel combustion, produced during anode baking processes, contain significant amounts of tar vapor and volatiles, which are problematic. The primary cast furnace produces exhaust gas flows and temperatures. The gas may contain hydrogen fluoride [27] at concentrations of less than 1 to 10 ppm, which limits the use of conventional heat exchangers for waste heat recovery. The melting furnace emits a considerable quantity of exhaust gases at temperatures around 870 °C, which may include flux material vapors, organic vapors, and particulates. An option is to treat the gases with absorbents, eliminating the need to cool the gases and producing cleaner gases suitable for heat recovery in existing systems.

The gases from crucible heaters, gas generators, and reverberatory (reverb) furnaces—with temperatures ranging from 790 to 1090 °C—contain natural gas combustion products and are free of major contaminants. These gases can account for up to 60% of the total heat input for heating systems and are viable candidates for heat recovery. Rotary furnaces produce cold gases laden with a large number of contaminants, and the industry has yet to develop an approach for their recovery. Consequently, research is underway to develop high-temperature polymers or composites for gas cleaning applications in waste heat recovery. Delacquering systems remove volatile materials, such as paint coatings, from used beverage cans and preheat them to about 480 °C before releasing them from a rotary kiln. The unit emits relatively clean exhaust gases at a temperature of about 340 °C, yet these gases do not undergo any heat recovery. Nonrecoverable heat is also found in crucible heaters. Nowicki et al. presented a mapping of heat demands at Alcoa Deschambault Quebec (ADQ) [76]. Their conservative assumptions indicated that the ADQ facility had the potential to produce approximately 10 MW of electricity using extractable waste heat. An aluminum foundry in Romania evaluated a waste heat recovery system [77]. Uses for the waste heat in the foundry included direct heating during winter, conversion to steam during summer, and electricity generation during spring and autumn when both heating and cooling demands are low. Wang et al. analyzed the performance of the organic Rankine cycle (ORC) in recovering low-temperature waste heat from aluminum reduction cells [78], observing that the variation in heat source temperature significantly affects net power output. When wind and solar power are used as a major energy supply for industrial aluminum electrolysis, the heat-exchanging system is also suitable for aluminum cells. Waste heat can be used for the aluminum die-casting industry [79]. Egilegor et al. analyzed the potential of heat recovery in aluminum low-pressure die casting to ensure effective operation and prolonged lifetime under high temperatures [80]. They concluded that given the working temperature/pressure and fluids, selecting appropriate materials for the heat pipe shells was crucial.

### 4.2. Aluminum Dross

**Figure 6:**
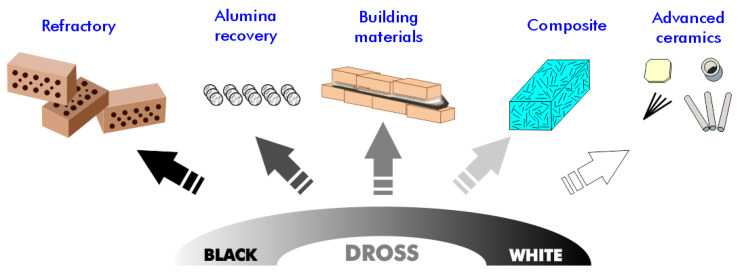
Potential application of aluminum dross residues.

Given the significant variability in the composition of aluminum dross among batches, greater effort is required to identify potential applications for this material. Various techniques for recycling and reusing aluminum dross show promise for managing aluminum dross and its reuse in diverse applications, such as the development of construction and building materials, separation media and agents, alumina extraction, and hydrogen production (Figure 6). We explore several of these applications in the following subsections.

#### 4.2.1. Aluminum Metal Recovery

**Figure 7:**
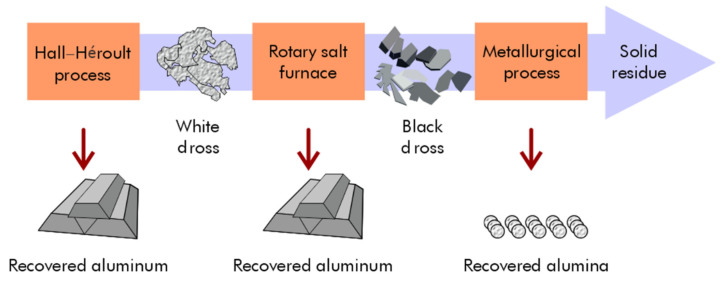
Recovery of aluminum and alumina from white and black dross using RSF and metallurgical processes.

Aluminum metal can be recovered using cost-effective recovery processes, and aluminum oxides find applications in the metallurgical and construction industries. The recycling and reuse of materials are crucial for reducing waste generation and decreasing the economy’s dependence on the extraction of primary (virgin) raw materials. This circular economy conserves resources and energy while slowing the depletion of virgin natural resources. During the Hall–Héroult process, aluminum metal can be reclaimed from white dross, which can be recycled through a rapid slurry-forming (RSF) process (Figure 7).

Several methods have been developed to recover dross while retaining the maximum amount of aluminum metal, such as spreading hot dross to accelerate cooling, combining dry pressing and alkaline roasting processes, and using an inert gas like argon to prevent oxidation [81]. The standard procedures for processing aluminum dross involve initially cooling the dross to room temperature using methods such as rotary drum coolers, stationary inert gas coolers, and floor spreading. The remaining metal can be recovered by reheating the raw dross (or a milled concentrate) with salt flux in a rotary furnace or, less commonly, in a side-well reverberatory furnace [82].

Manual separation and density-based separation methods like hydraulic and pneumatic classifiers are less effective. Zuo et al. demonstrated a method for recovering salts and ammonium hydroxide from black aluminum dross using catalytic hydrolysis, followed by filtration and drying before calcination [83]. Aluminum and other valuable elements can be recovered in the form of alloys, such as aluminum nitride (AlN), by adding alumina in electrochemical reduction. Salts are also reclaimed during the filtration of leachate followed by crystallization.

#### 4.2.2. Applications in Construction Materials

**Figure 8:**
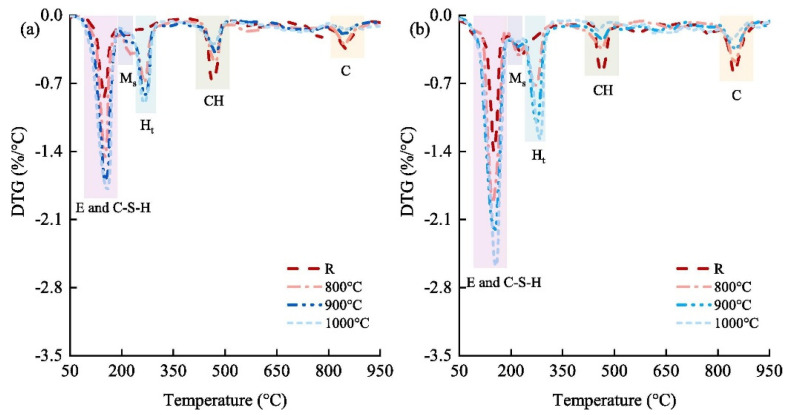
Differential thermogravimetry (DTG) of pastes at curing times of (**a**) 3 days and (**b**) 28 days. E, ettringite (Ca_6_Al_2_S_3_H_64_O_60_); C-S-H, calcium silicate hydrate; Ms, monosulphate (Ca_6_Al_2_SH_24_O_22_); Ht, hydrotalcite (Mg_6_Al_2_CH_24_O_52_); CH, portlandite (CaO_2_H_2_) [85].

The use of white or black dross as a filler in the construction industry is common. Both white and black dross can serve as fillers in asphalt—when the particles are smaller than 700 μm—to improve stiffness and abrasion resistance and reduce microcracking [84]. Zhang et al. developed novel supplementary cementitious materials (SCMs) by mixing secondary aluminum dross with dolomite [85]. They investigated the effect of thermal activation on calcined mixes, the hydration properties of pastes containing novel SCMs, and the workability of cemented paste backfill (CPB). High-temperature calcination significantly increased SCM activity, and thermal activation enhanced the activity of SCMs (Figure 8).

**Figure 9:**
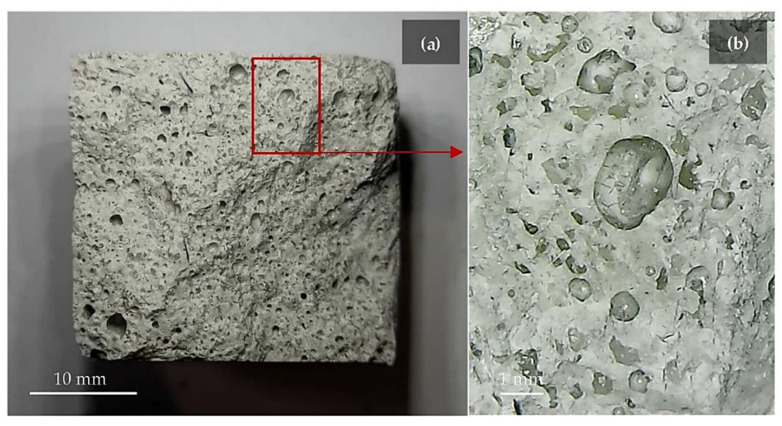
(**a**) Geopolymer foam section and (**b**) magnified image of bubbles generated by the oxidation of the by-product [86]. The gas bubbles were generated during the consolidation process of by-product and alkali activator.

The black-dross-leached Al residue, produced during the hydrothermal treatment of Al black dross, can also be used as a raw material for producing Portland cement clinker [85]. Ercoli et al. evaluated the mechanical and thermal properties of geopolymer foams reinforced by carbon fibers, using by-products from the secondary aluminum industry [86]. The flexural and tensile strengths decreased when aluminum-rich by-products were added as additives to foam the geopolymers because of the gas bubbles formed in their structure during the consolidation process (Figure 9 illustrates the gas bubbles in the geopolymer foam, which are not homogeneous in size). However, the Charpy impact strength value was increased because chopped carbon fibers reinforced the geopolymer.

#### 4.2.3. Dross for Engineering Composites

**Figure 10:**
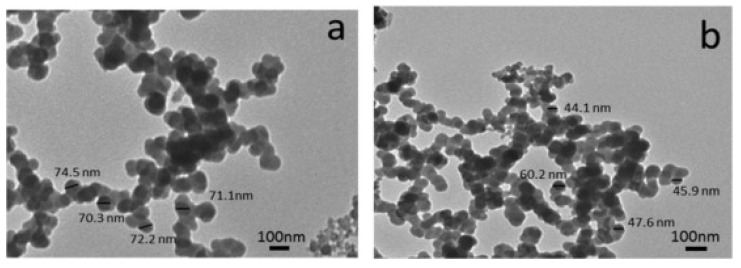
TEM images of (**a**) Al-dross waste powder without reinforcement and (**b**) W15 powder that contains 7.5 wt% SiC and 7.5 wt% TiO_2_ [89].

Dirisu et al. used uncarbonized egg shell [87] to reinforce aluminum dross, cement, silicate, and pulverized carbon graphite. They applied a molding process to produce a building ceiling composite and investigated how its composition affected thermal conductivity and microstructure. The sample comprised 30 wt% Al dross, 25 wt% cement, 30 wt% silicate, 5 wt% carbon graphite (CG), and 10% uncarbonized eggshell (UES). The composite could serve as a flame-retardant ceiling because of its lower thermal conductivity relative to existing ceiling tiles. Moreover, the produced composite exhibited a lower heat flux than other previously developed ceiling composites. The production of conventional materials from expensive raw materials is increasingly being replaced by more cost-effective composite materials [88]. Dross, SiC, and TiO_2_ nanopowders have been analyzed for use in ceramic composites [88]. SiC is known for its desirable properties, including high hardness, strength, and melting point; good chemical and thermal stability; and oxidation and erosion resistance. TiO_2_ can be added to SiC with the aim of improving the sinterability and properties of the composites [89]. TEM images of sintered composites, with and without added SiC and TiO_2_ (after being milled for 5 h), show no particle coarsening, and the particle size of the waste Al dross decreases as the weight percent of SiC and TiO_2_ (as reinforcement) increases (Figure 10).

#### 4.2.4. Other Applications

**Figure 11:**
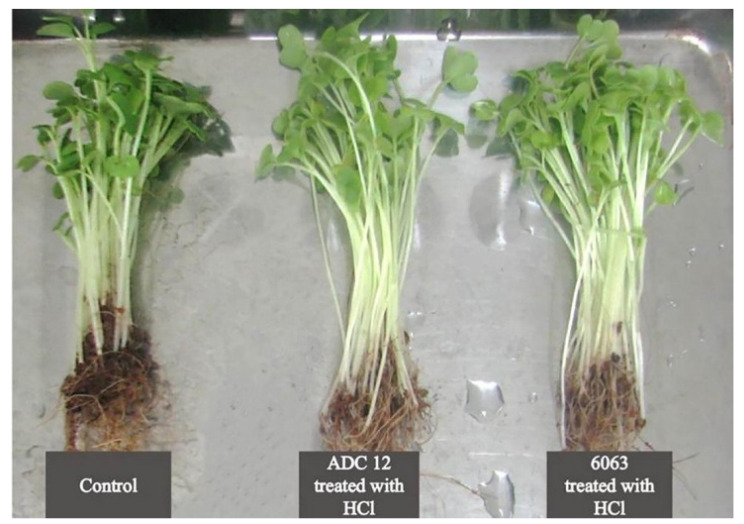
Untreated and dross-treated Chinese cabbage after 13 days [94]. The plants showed increased height and weight compared to the control plants.

Other uses of aluminum dross include its application in the steel, ceramic, and fertilizer industries. Heo and Park investigated Fe recovery from electric arc furnace (EAF) slag using aluminothermic smelting reduction (ASR) at 1773 K with Al dross as the reductant [90]. During the FeO reduction reaction, solid spinel (MgO·Al_2_O_33_) and MgO compounds precipitate from the slag. Moreover, the metallic Al in the Al dross reductant can reduce Mn from the EAF slags. The use of black dross to produce a ladle-fluxing agent for the steel industry has been studied [91]. Ewais and Besisa produced magnesium aluminate titanate (MAT)-based ceramics by sintering aluminum dross waste and combining it with rutile ore powders at 1300 °C for 6 h [92]. The obtained samples were thermally stable and did not decompose at high temperatures, presenting a promising method for producing a new advanced ceramic material. Zhang et al. synthesized MgAl_2_O_4_ spinel from secondary aluminum dross [93]. They doped the spinel with rare earth oxides (REO), including Y_2_O_3_, Eu_2_O_3_, La_2_O_3_, and CeO_2_, to improve the densification behavior. The densification of the MgAl_2_O_4_ doped with lanthanum oxide was higher when heated at 1473 to 1773 K Dangtungee et al. neutralized aluminum dross and used it as fertilizer [94]. The treated aluminum dross fertilizer enhanced the height and weight of Chinese cabbage relative to untreated plants and controls (Figure 11). Sweet corn, basil, and spring onions also exhibited improved growth with the aluminum treatment. Because no toxic heavy metals are present in the prepared fertilizer, aluminum dross—after neutralization—can be considered an environmentally friendly fertilizer.

Aside from metals and oxides, gaseous phases such as NH_3_ can be extracted during the synthesis of materials from aluminum dross. Gomes et al. used ammonia (NH_3_) released from Al dross as a nitrogen source for sprouting seeds [95]. Vegetable and fruit seeds were planted in soils directly infused with dross-produced NH_3_ for 2 h, followed by 8 to 12 h of self-diffusion time to ensure even distribution of the gas throughout the soil. Seeds that prefer acidic soils did not germinate well in soils infused with NH_3_, whereas seeds that thrive in alkaline conditions, such as ridge gourd and watermelon seeds, sprouted early and robustly. Shi et al. [96] created a polyaluminum chloride flocculant by treating secondary aluminum dross with hydrochloric acid. The new flocculant effectively removed impurities from wastewater and other waste liquids. Philipson et al. [97] produced a silicon alloy reducing silica in calcia–silica (CaO-SiO_2_) slag with aluminum dross as a reductant. The kinetics of reduction were investigated by comparing the Si-alloy and product slag compositions to simulated thermodynamic equilibrium. The Al dross was shown to be as effective a reductant as pure Al, and the results show rapid aluminothermic reduction and good Si-alloy/slag separation for both reductants.

### 4.3. Salt Slag

**Figure 12:**
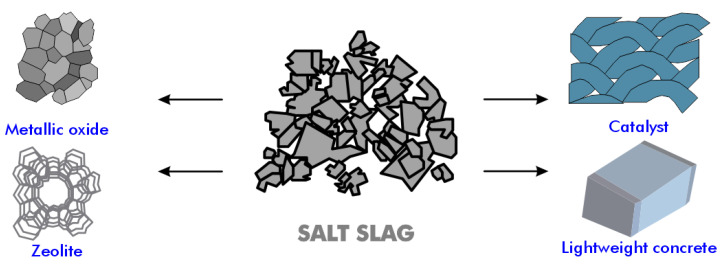
Various applications of aluminum salt slag.

Salt slag, a material produced during the melting of aluminum scrap or dross, contains aluminum, aluminum oxide, alkaline chlorides, and impurities such as carbides, nitrides, sulfides, and phosphides [98,99]. The treatment of salt slag is typically carried out in the US, Canada, and Europe, where the landfilling of salt slag is prohibited by law. The treatment of salt cake results in a significant financial return. The salt fraction, including halite (NaCl) and sylvite (KCl) along with residual metallic aluminum, can be recovered, justifying the investment in salt cake recycling facilities by large refining companies [100]. The residues generated post-recycling are non-toxic, and the alumina-containing compounds can be used as new raw materials in other processes and applications, such as in the cement industry and as refractory materials (Figure 12).

#### 4.3.1. Synthesis of Zeolite

**Figure 13:**
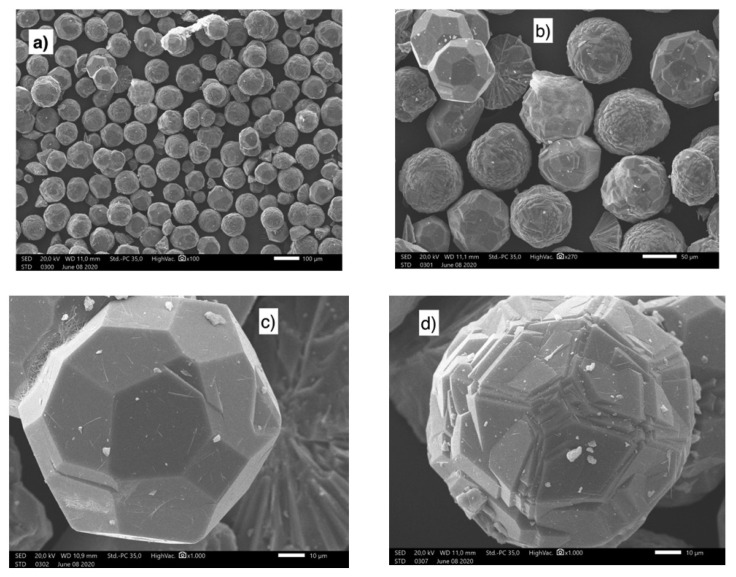
SEM images at different magnifications of ANA zeolites obtained from the aluminum waste [102]. (**a**,**b**) demonstrated the existence of particles with a diameter of less than 100 μm. (**c**,**d**) showed pentagonal faces zeolite.

Sánchez-Hernández et al. used hydrolyzed salt as a reactant along with Na_2_SiO_3_ and NaOH solutions to synthesize zeolite [101]. Their single-step method produced zeolite-type NaP (Na_6_Al_6_Si_10_O_32_·12H_2_O) by adding the corresponding amount of silicate solution. The produced zeolite exhibited characteristics similar to those of conventional chemical reagent–based forms. Jimenez et al. synthesized pollucite and analcime zeolites by extracting aluminum from saline slag [102]. The structures of the synthesized materials are based on (Al,Si)O_4_ tetrahedra that share corners and feature pores, channels, and/or cavities at the molecular level. The extraction liquor contained aluminum and alkali metal cations. After adding the required amount of Si and undergoing hydrothermal synthesis at 200 °C for 24 h, zeolitic materials were produced (Figure 13 shows SEM images of this material). The crystallinity and water content of analcime were higher than those of pollucite. Morphological analyses revealed the formation of spherical particles, which are larger for analcime solids with pentagonal or polygonal faces. For pollucite, spheres are smaller, and it is possible that they are hollow.

#### 4.3.2. Application in Construction Materials

Lin et al. explored the use of recycled aluminum salt slag (RASS) as aggregate combined with recycled concrete through the alkali-activation method for stone column materials [103]. Both materials were stabilized by fly ash and ground granulated blast furnace slag to improve soft soils. Increasing the RASS content decreased the unconfined compressive strength [104] of the mixtures; however, the mixture of recycled concrete aggregate with 5% alkali-activated fly ash and slag met the minimum unconfined compressive strength [104] requirement for ground improvement.

### 4.4. Spent Carbon Cathode

**Figure 14:**
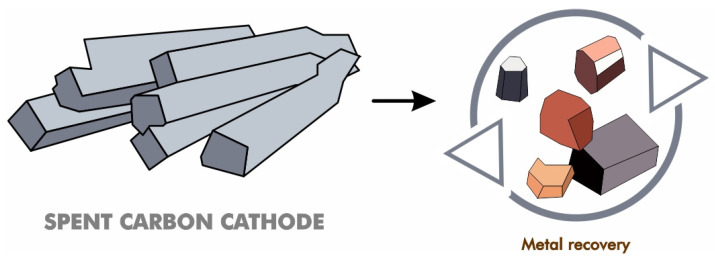
Recovery of various metal from spent carbon cathode.

Spent carbon cathode is considered as hazardous solid wastes from aluminum production. Therefore, studies are carried out to investigate cost-effective and environmentally friendly purification techniques for separating carbon and subsequently using it efficiently. Most of the research is on metal recovery (Figure 14), which will be elaborated on in the next subsection. Additionally, the use of spent carbon cathode for other applications will also be discussed.

#### 4.4.1. Metal Recovery

Carbon recovery from spent carbon cathode is studied by several researchers. For example, Yuan et al. [105] synthesized silicon carbide with silicon dioxide at 1600 °C, giving the spent cathode carbon regeneration value, and obtained high-purity carbon powder through ultrasonic leaching. High-purity recovered carbon was also obtained from the research of Lu et al. [106] to be used directly as the anode for Na-ion batteries in order to benefit from these characteristics and reuse the high value-added carbon material. The capacitive nature of Na^+^ in recovered carbon material allows the electrode to demonstrate notable electrochemical performance in the electrolyte solvent. However, there is a challenge of physical leaching treatments that produced low carbon extraction [107]. Other metals, such as iron and silicon, have been recovered from spent carbon cathodes for the treatment of red mud [108]. Fluoride can also be recovered from spent carbon cathodes. Robshaw et al. [109] used chelating resin loaded with lanthanum ions. The resin showed great promise in recovering fluoride from aqueous waste streams and demonstrated a large defluoridation capacity.

#### 4.4.2. Other Applications

A hydrothermal acid-leaching technique was employed by Xiao et al. [110] to separate the various components in spent carbon cathode. While carbon and silicon remained in the filter residue, impurity elements like Al, F, and Fe entered the solution. Additionally, they used the carbothermal reduction method to create silicon carbide products. Spent carbon cathode has a highly expanded graphitic structure by nature [111]. This can be advantageous in preparing the anodes of lithium-ion batteries [112]. The anode material used was the purified graphitized spent carbon cathode which has better cycle stability. Carbon anodes for aluminum electrolysis were made by Yao et al. [113] using the recovered carbon material from roasting molten salt. It was performed better compared to carbon anodes made entirely of petroleum coke. This suggested that SCC might be utilized as raw materials to make carbon anodes for aluminum electrolysis. Zhi et al. [114] treated the spent carbon cathode with water washing process, and recovered large amount of sodium fluoride (NaF). Li et al. [115] investigated the thermal behavior of spent carbon cathode block blended with molten salts. It was discovered that NaCl-Na_2_CO_3_ binary molten salts were efficient in lowering the fluoride content of spent carbon cathode blocks and preserving the energy needed for the thermal treatment procedure.

### 4.5. Bauxite Residue

The proposed solution to bauxite residue stockpiling involves creating comprehensive use technology or transforming it into a secondary resource. Since the 1950s, multiple research projects have focused on the disposal and use of bauxite, given its distinctive physical and chemical characteristics, such as high pH and the presence of trace amounts of heavy metals, which can pose a serious environmental risk [116]. The production of cleaner bauxite residue is now a primary focus for the aluminum industry. The subsequent subsections will discuss the waste management of bauxite residue, neutralization of impurities, extraction of metal oxide and rare earth elements and the use of this residue in environmental, energy, coating, construction, and geopolymer applications.

#### 4.5.1. Waste Management Methods

Wet processing, dry processing, and semi-dry processing are the three standard methods for disposing of bauxite residue in landfills [117,118,119]. During wet processing, bauxite residue is first washed in a thickener to facilitate the sedimentation of solids and produce a residue slurry with a solid content of 15% to 30%. The resulting slurry is then pumped into a storage yard comprising earthworks, tailing ponds, and dams to allow the solids to settle. The liquid supernatant in the storage yard is collected and used to generate recovered caustic soda [120]. However, the disposal of the dilute residue slurry after wet processing poses environmental risks to nearby areas/communities because of the potential release of caustic and highly alkaline (pH >12) liquid into the subsoil water [121].

The dry-stacking method and dry cake disposal have been adopted by many aluminum production plants because of their reduced spatial footprint and lower potential for leaks, as landfills are becoming scarcer and space-limited [122]. In this process, the bauxite residue is pre-thickened to produce a residue slurry with a solid content of over 50%, which is then deposited in layers ranging in thickness from 0.4 to 0.7 m. Drum filters or plate and frame filter presses are used to achieve the high solid content of the residue slurry during filtration. Bulldozers turn up the bottom layer to expedite drying and minimize dust emissions. Successive layers of residue slurry are placed on top to expand the spatial storage capacity, thereby reducing the landfill space occupied by bauxite residues [123].

Semi-dry processing refers to the co-disposal of bauxite residue slurry generated by the Bayer and sintering processes. The residue typically exhibits a low permeability coefficient and high shear yield strength because of its hydration and hardening capabilities [124]. Thus, the dried residue from the sintering process can directly replace clay in the construction of dams.

#### 4.5.2. Neutralization of Bauxite Residues

Efforts have been made to partially neutralize bauxite residues to mitigate the environmental impact of their high alkalinity. These approaches include seawater neutralization for plants near the sea, carbonation treatment, acid neutralization, sintering, and bioleaching. The addition of seawater to caustic red mud decreases the pH, leading to the precipitation of minerals such as hydroxycarbonate, carbonate, and hydroxide [125]. Seawater neutralization converts readily soluble, strongly caustic wastes into less soluble, weakly alkaline solids, although it does not completely eliminate hydroxide. The waste’s carbonate and bicarbonate alkalinity are primarily removed through reactions of hydroxide with calcium to form aragonite and calcite [126]. Neutralization is considered complete once the liquid from the treated bauxite residue has a pH below 9.0 and a total alkalinity of less than 200 mg/L (measured as calcium carbonate equivalent alkalinity), allowing the decantation of seawater-neutralized bauxite residue to be safely discharged into the marine environment [127].

Red mud neutralization by carbon dioxide gas has been studied [104]. Gaseous CO_2_ or flue gas containing CO_3_^2−^ is bubbled through aqueous slurries to form carbonic acid in the aqueous phase [128]. This carbonic acid reacts with the basic components of red mud, thereby lowering its pH. However, only a fraction of the alkaline material in red mud is neutralized by gaseous CO_2_ within the short contact times necessitated by industrial process rates. Consequently, although the pH of the aqueous phase decreases rapidly upon exposure to CO_2_, it quickly returns to unacceptable levels as more alkaline material leaches from the mud. Given that the pH of water exposed to gaseous CO_2_ is unlikely to fall below approximately 5.5, the neutralization rate of the solids in the aqueous slurry typically does not meet industrial requirements. Researchers have, therefore, explored the use of high-pressure liquid CO_2_ instead of its vapor phase for more effectively reducing the pH of red mud [129], including the approach of neutralizing red mud with CO_2_ over multiple cycles [130].

A variety of aqueous acidic solutions, including acidic industrial wastewater, have been considered for neutralizing residual alkalinity. Several studies have assessed the feasibility of treating bauxite residues, such as the Kwinana red mud slurry, with acid [131]. However, the complete neutralization of the residue requires large volumes of reagent, even when using spent (waste) acid, leading to relatively high costs. Additionally, acid treatment introduces significant quantities of impurities into the process’s water stream, such as sulfate in the case of sulfuric acid and chloride in the case of hydrochloric acid. Therefore, reintroducing any water from the residue deposits back into the production process is likely to be unacceptable without further treatments to eliminate these added impurities. The treatment of red mud with acidic spent pickling solutions (SPSs) derived from the steelmaking process produces a coagulant—a mixture of aluminum and iron salts—for wastewater treatment. Although sintering the residue can control all leachable soda, this process can be prohibitively expensive given the increased energy consumption required for high-temperature sintering of red mud. Alcoa of Australia has implemented a bioremediation of bauxite residue in Western Australia by adding organic substrate to the red mud to cultivate microorganisms. These microorganisms produce various organic acids and, in some cases, CO_2_, which neutralizes the mud [131].

Efforts to find viable solutions for bauxite residue disposal continue, including efforts to improve storage, monitoring, and safety standards to reduce environmental impacts, develop techniques to enhance storage yard reclamation potential, and promote reusability methods to decrease the volume of accumulated bauxite residue [132]. Despite these efforts, red mud still occupies a significant amount of landfill space, and sustainable waste management strategies are needed to use red mud as a secondary resource material.

#### 4.5.3. Extraction of Metal Oxides from Bauxite Residue

**Figure 15:**
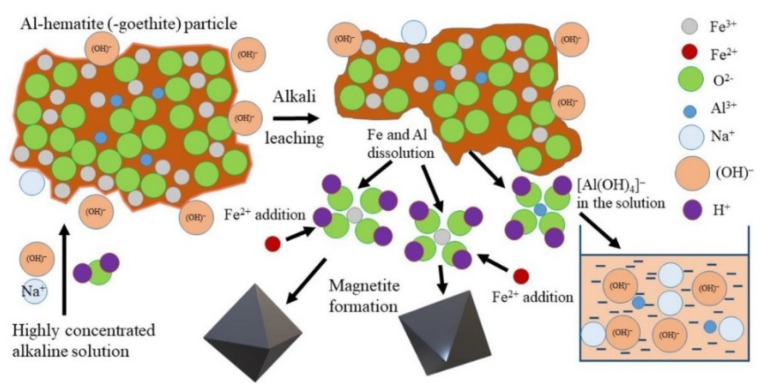
The schematic mechanism of high-iron bauxite residue leaching by a highly concentrated NaOH solution in the presence of Fe^2+^ [133].

Bauxite residue primarily consists of Fe_2_O_3_, Al_2_O_3_, SiO_2_, TiO_2_, Na_2_O, and CaO (Table 1). Various methods exist for extracting these oxides. The high Na_2_O content limits the potential application of red mud for iron-containing products, as it is not easily soluble in water, resulting in high alkalinity. However, the hydrochemical conversion of goethite to magnetite has been proposed as a solution to this issue [133]. High-iron bauxite residue leaching can occur in the presence of Fe^2+^ (Figure 15), as this ion facilitates the extraction of Al from Al-hematite and Al-goethite via the precipitation of magnetite from the solution. The simultaneous extraction of Al and Na results in a product containing more than 96% Fe_3_O_4_.

Li et al. proposed a method for extracting Al_2_O_3_ and TiO_2_ from red mud smelting separation slag through mineral phase reconstruction conducted under an air atmosphere [134]. Al_2_O_3_ and SiO_2_ are converted into alkaline-soluble NaAlO_2_ and Ca_2_SiO_4_ by alkaline hydroxide roasting. The resulting NaAlO_2_ solution can serve as a source for extracting alumina, with more than 80% of Al_2_O_3_ selectively dissolved in a 95 °C NaOH leaching solution. Subsequently, SiO_2_ is extracted from the residue using an HCl solution, yielding a solution containing SiO_2_ and a concentrated residue of undissolved CaO and TiO_2_. Hydrochloric acid leaching was used to recover the latter.

A novel process for the coupled treatment of coal gangue and red mud has also been developed [135]. Guo et al. demonstrated that red mud can adjust the Al/Si molar ratio of coal gangue to an appropriate level and that the Na_2_O in the red mud can partially substitute for the Na_2_CO_3_ required for coal gangue activation, thereby reducing the amount of Na_2_CO_3_ consumed from 100% to 12.1–20.5%.

#### 4.5.4. Extraction of Rare Earth Metals from Red Mud

**Figure 16:**
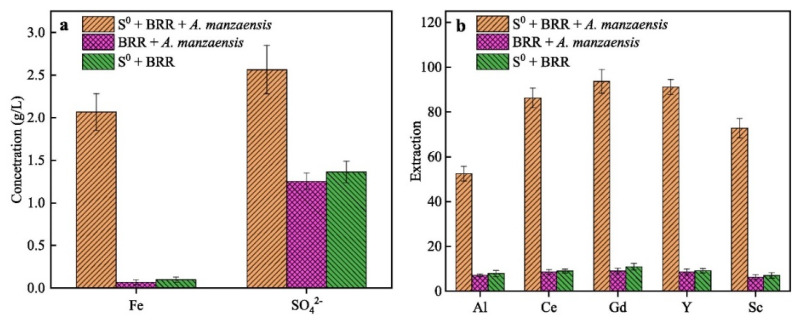
(**a**) [Fe^2+^] and [SO_4_^2−^] concentrations and (**b**) the extraction rates of Al, Ce, Gd, Y, and Sc during the anaerobic bioleaching of red mud using *Acidianus manzaensis* [140].

In addition to the extraction of metal oxides from red mud, several rare earth elements (REEs) may also be extracted [136]. Small amounts of Sc, Y, La, Ce, Pr, Nd, Sm, Eu, Gd, Tb, Dy, Ho, Er, Tm, Yb, and Lu are found in red muds [137]. Red mud has been identified as one of the promising sources of scandium (Sc) [138]. Dai et al. developed a low-cost and environmentally friendly biosorbent for Sc extraction [139]. In their study, H_3_PO_4_-activated biochar (P40s) produced from pitaya peels was used for Sc adsorption and recovery from red mud. This biochar demonstrated optimal reusability after five adsorption/desorption cycles. The extraction of Al and REEs from red mud via aerobic and anaerobic bi-stage bioleaching has also been explored [140] by Zhang et al., which achieved the highest extraction rates for Al, Ce, Y, and Sc (Figure 16). Thus, the release of valuable elements from bioleached red mud residue can be enhanced by bio-oxidation under anaerobic conditions.

Roasting can also remove calcium and obtain rare earth element (REE) oxides from red mud; this process is followed by the selective mixing of sulfuric acid with REE oxides. Calcium, aluminum, and iron components can also be recovered at each stage [141].

#### 4.5.5. Bauxite Residues for Environmental Applications

Carbon capture and storage (CCS) technologies are a significant area of research aimed at reducing greenhouse gas emissions. Direct mineral carbonation is a CCS technology that offers the benefits of permanence, affordability, and promising on-site options, while also enhancing the environmental quality of waste. In recent years, red mud has been explored for gas cleaning treatments.

**Figure 17:**
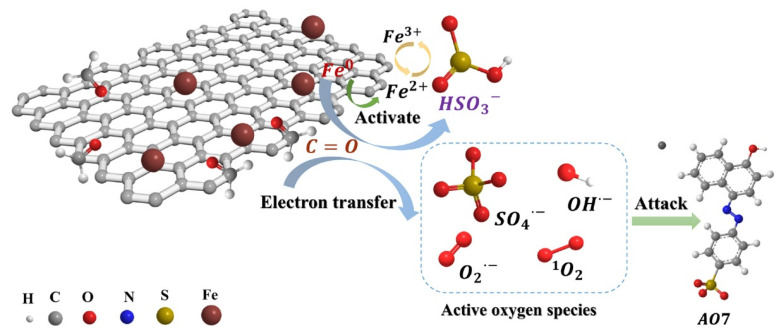
Acid orange 7 (AO7) degradation in the biochar composites [143]; both radical and nonradical processes broke down the adsorbed AO7 molecules. Notably, SO_4_^2−^ played a significant part in the degradation process.

The use of red mud in reducing concentrations of residual antibiotics in composting has been investigated [142]. Composting has been found to decrease the concentrations of most residual antibiotics, as well as the abundance of antibiotic resistance genes and mobile genetic elements. In another application, Li et al. synthesized zero-valent iron (ZVI) biochar composites from red mud and ginkgo leaves [143]. Ginkgo leaves contain active groups that have a strong adsorption effect on heavy metal ions [144]. Iron phases in red mud were transformed from Fe_2_O_3_ to Fe^0^ after pyrolysis at 800 °C with a red mud:ginkgo leaf mass ratio of 2:1; the iron phase and ketonic functional groups (C=O) were identified as two key active sites (Figure 17). The synthesized composites were effective in removing bisulfate and acid orange 7.

Bai et al. proposed the use of red mud for purifying heavy metal pollutants in water [145]. The adsorption strength for Pb, Cd, and Cu can be enhanced by increasing the liquid temperature. Guo et al. synthesized biochar-supported red mud catalysts for the degradation of COVID 19–related drugs such as arbidol and acyclovir [146]. The presence of functional groups in red mud catalysts, which contain oxygen and Fe species (Fe^0^ and Fe_3_O_4_) as well as Ca^2+^ ions, was crucial for the removal of arbidol.

**Figure 18:**
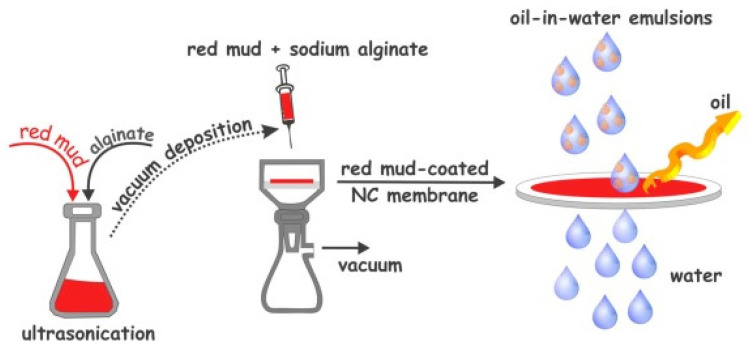
Schematic illustration of the fabrication of red-mud-coated-nitrocellulose (NC) membrane for oil-in-water emulsion separation [147].

The efficient treatment of industrial oily wastewater before discharge into receiving bodies is critical for preventing environmental pollution and protecting human health. Membranes with superwettable surfaces have been regarded as promising materials for the separation of oil/water emulsions [147]. These surfaces include nitrocellulose membranes coated with red mud (Figure 18).

#### 4.5.6. Bauxite Residue as Construction Material

**Figure 19:**
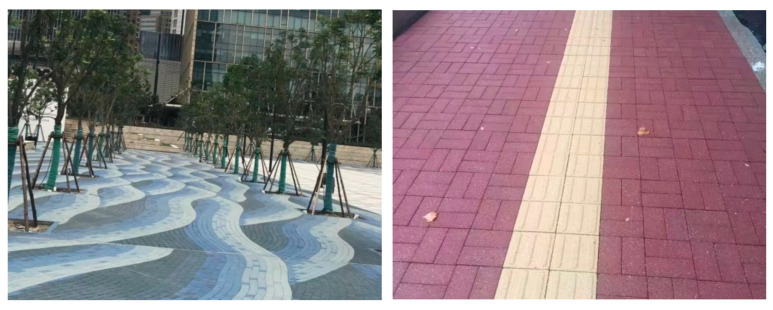
Red mud–based permeable brick produced in Shandong Province, China [150].

Portland cement is the most widely used type of cement for general construction purposes. Most CO_2_ emissions from the cement industry occur through the process of decarbonizing limestone during the creation of clinker. Given the rising demand for infrastructure and sanitation, the primary method for reducing cement use (and thus greenhouse gas emissions) in the civil construction sector involves the partial or complete replacement of Portland cement clinker. Several studies have explored producing various types of cement from red mud [148,149]. The moderate amounts of SiO_2_ and Al_2_O_3_ in red mud allow for its potential use as a material for mortar, concrete, bricks, aggregates, and roofing. Zeng et al. discussed the production of a permeable red mud–based brick using a fully automatic production line for ecological RM permeable bricks (Figure 19) [150]. The company’s daily output was 3000 m^2^, and its production process could digest nearly 100,000 t of RM annually. The brick exhibited a bending strength of 6–8 MPa, exceeding the national standard of 3.2 MPa, with an RM content of up to 80%.

Wang et al. incorporated red mud into road cement clinker [151]. For developing Portland cement for road clinker, they first de-alkalized the sintering red mud and then combined it with materials such as clay, fly ash, limestone, and sandstone. Each group of raw materials containing red mud exhibited a lower thermal decomposition temperature than the control materials; this reduction in decomposition temperature related to the accelerated carbonate decomposition process of the raw materials for roads facilitated by the addition of red mud.

**Figure 20:**
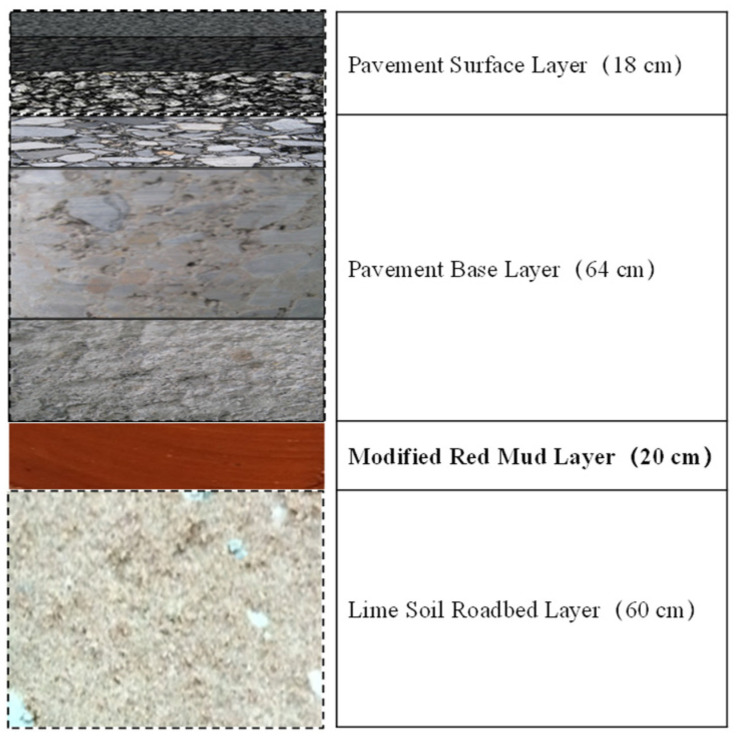
Pavement structural diagram of a demonstration road in Jinan Qingdao Highway, China [153].

The structural characterization of red mud in roof tile manufacturing has also been investigated [152]. Increases in both firing temperature and RM content enhance the compressive strength of fired samples. Ma et al. studied modified Bayer red mud for use as a highway subgrade and assessed its performance in a large-scale application as part of a road trial for the Ji-Qing Highway Reconstruction and Expansion Project in China [153]. The emplaced subgrade spanned approximately 4900 m in length, 11 m in width, and 0.2 m in thickness (Figure 20). The modified red mud replaced the standard 0.2 m of lime-stabilized soil in the upper part of the roadbed. This modified Bayer red mud showed significant improvements in road performance. The pH of the leaching solution decreased, and the compressive strength reached up to 3 MPa within three days. Additionally, the concentration of the leached harmful components decreased by more than 70% relative to the pretreated red mud.

**Figure 21:**
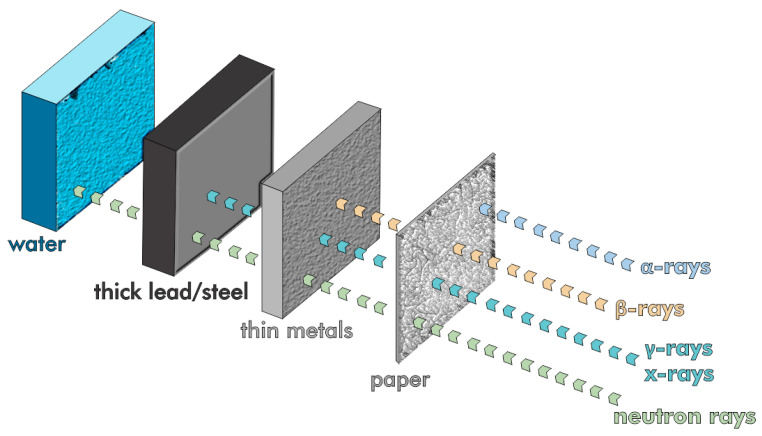
The comparative penetrative power of various radiation sources.

Iron and other high-atomic-number (Z) waste materials, such as steel slag and red mud, can be used to create X- and γ-ray shielding concrete [154,155]. Human skin itself blocks alpha particles, whereas beta radiation is stopped by thin sheets of wood or aluminum (Figure 21). The most challenging forms of radiation to attenuate are highly penetrative and dangerous uncharged gamma and neutron radiation.

Aside from concrete, lead is often used to shield against high-energy photons given its high atomic number and density [156]. The challenge with radiation shielding in concrete is predicting radiation attenuation because of variable moisture within the concrete. Although commonly used, lead poses significant health and environmental risks, and its heterogeneous nature complicates predictions of its effectiveness as a radiation barrier [157,158].

Agrawal et al. fabricated diagnostic X-ray shielding tiles from red mud and barium sulfate (BaSO_4_) with the addition of sodium hexametaphosphate (SHMP) and kaolin clay to enhance their mechanical strength [159]. The radiation attenuation of the tiles, measuring 10.3 mm and 14.7 mm in thickness, was equivalent to that of 2 mm and 2.3 mm of lead at 125 and 140 kilovoltage peak (kVp), respectively. These tiles are already being used in bone densitometry and catheterization laboratories.

#### 4.5.7. Bauxite Residue for Geopolymers

**Figure 22:**
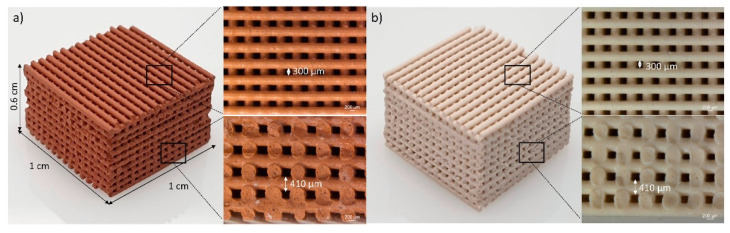
Optical micrographs of 3D printed red mud with (**a**) 50% red mud and (**b**) 100% metakaolin [163].

Geopolymer is an inorganic polymer material composed of aluminosilicate and an alkali activator. The sources of Al_2_O_3_ and SiO_2_ can be derived from natural minerals such as metakaolin and clay, given their abundance in many regions of the world [160]. The drive for more eco-friendly materials has spurred interest in using this metal oxide-rich industrial waste. Such residual materials include fly ash, silica fume, blast furnace slag, and air-cooled slag [161,162]. Gonçalves et al. produced 3D-printed red mud/metakaolin-based geopolymers as methylene blue sorbents from water pollutants (Figure 22) [163]. Lattices with approximately 62% open porosity were achieved, showing no significant adsorption loss after 10 cycles of operation. These geopolymer composites exhibited low leachability with regard to contaminants, especially those with notable compressive strength. The development of geopolymer-based protective coverings has recently garnered increased attention. Fly ash–red mud geopolymers have been explored as coating materials for mild steel [164], with the developed material providing excellent corrosion protection, thereby helping to extend the service life of structures made of mild steel.

**Figure 23:**
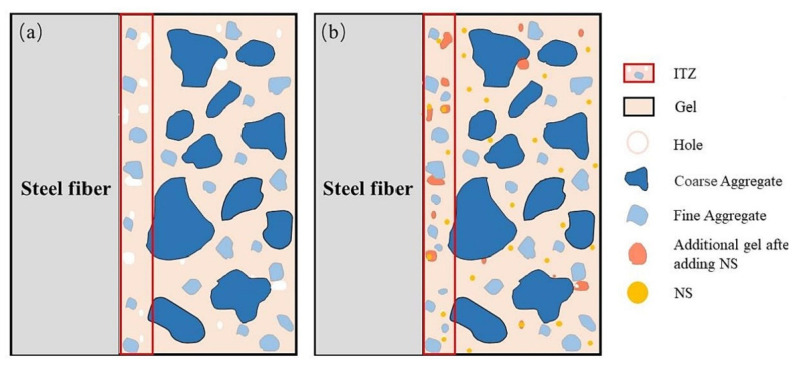
A nano-SiO_2_-reinforced fiber matrix interfacial transition zone (ITZ); (**a**) without nano-SiO_2_ (NS) and (**b**) with NS [165].

Xu et al. investigated the effects of nano-SiO_2_ (NS) and steel fiber on the properties of red mud–based geopolymer concrete [165]. They found that steel fibers and nano-SiO_2_ significantly enhanced the performance of the red mud geopolymer composite, with the most substantial improvements observed when both nano-SiO_2_ (NS) and steel fiber were combined. The interface transition zone of the NS-reinforced fiber matrix is depicted in Figure 23.

The thermal-related performance of PCM (phase change material) geopolymer composites has also been evaluated [166] by Afolabi et al., which used expanded graphite (EG) to encapsulate PCMs as a composite, then vacuum-impregnated the composite into geopolymer aggregates to create an enhanced thermal wall material for use in buildings and construction. The outdoor thermal storage performance of the EG/PCM composite geopolymer wall material exceeded that of conventional materials such as cement, clay, and gypsum.

## 5. Conclusions

Despite recent technological advances, the disposal of aluminum dross, salt slag, and bauxite residue remains a challenge for the aluminum industry, with the problems with resource utilization of these residues and critical issues continuing to impact the feasibility of recycling and the economics of the process. Multiple applications must be implemented to reduce the volumes of generated waste. In this review, we discussed the use of these residues in several construction products, including their utility as materials for cement and concrete, as supplemental additives to Portland cement, and as geopolymers to replace cement materials. The valorization of this industrial waste requires controlled processing parameters to ensure that the finished products meet existing application standards. The effectiveness of aluminum industrial waste in new applications must be comparable to alternatives, such as steel and energy plant industrial waste, in terms of quality, price, and environmental risk. The aluminum industry must collaborate with aluminum associations and research institutions to develop and promote the potential use of residues and their large-scale reuse in a variety of applications.

## Figures and Tables

**Table 1:** Oxide compositions of red mud produced globally.

Country	Plant	Location	Major Constituents (wt%)						Ref.
			Fe_2_O_3_	Al_2_O_3_	SiO_2_	TiO_2_	Na_2_O	CaO	
Australia	Boyne Smelters	Gladstone	23–36	20–30	13–17	4–8	5–11	1–3	[51]
	Rio Tinto	Gove	30	20.8	17.1	8.3	8.1	2	[51]
	ALCOA	Kwinana	27–31	12–20	27–54	1–3	1–3.3	0–3	[51]
Brazil	Alunorte	Barcarena	45.6	15.1	15.6	4.29	7.5	1.16	[52]
Bosnia	Zvornik	Birac	48.5	14.14	11.53	5.42	7.5	–	[53]
Canada	Rio Tinto	Vaudreuil	31.60	20.61	8.89	6.23	10.26	1.66	[54]
China	Weiqiao	Shandong	59.37	16.16	9.11	–	2.78	2.17	[55]
France	Pechiney	Gardanne	42	14	6	11	2	–	[56]
Germany	FRG Baudart	Stade	38.75	20	13	5.5	8.16	–	[57]
Greece	Aluminum of Greece	Agios Nikolaos	44.60	23.6	10.2	5.7	2.5	11.2	[58]
Hungary	MAL	Ajka	38.75	15.2	10.15	4.6	8.12	–	[57]
India	Nalco	Damanjodi	47.85	22.64	12.51	3.58	10.25	1.86	[59]
Indonesia	ICA	Tayan	35.10	29.5	2.96	–	4.89	0.18	[60]
	BAI	Bintan	44.66	28.87	20.21	3.03	–	0.28	[61]
Iran	Jajarm	North Khosaran	22.17	13.98	13	7.17	2.01	24.25	[62]
Ireland	RUSAL	Askeaton	47	17	7	12	5	–	[63]
Italy	Eurallumina SpA	Sardinia	18	26	20	6	12	6.7	[64]
Jamaica	ALPART	Nain, St. Elizabeth	50.9	14.2	3.4	6.87	3.18	–	[57]
Russia	Bogoslovsky	Urals	42.1	12.7	9.4	4.3	4.8	7.8	[65]
	RUSAL	Kamensk-Uralsky	12.2	25.5	2.5	-	28.3	2.5	[66]
	Ural	Kamensk-Uralsky	36.9	11.8	8.71	3.54	0.27	23.8	[67]
	Tatarka Deposit	Krasnoyarsk Krai	6.86–34.05	41.44–59.45	1.48–7.97	1.17–4.08	0.05–0.39	0.01–0.4	[68]
Suriname	ALCOA	Paranam	24.81	19	11.9	12.15	9.2	–	[57]
Spain	Alcoa	Guinea	37.5	21.2	4.4	11.45	3.6	5.51	[52]
Taiwan	Sigma Group	Kaohsiung	41.3	20.21	17.93	2.9	3.8	–	[57]
Turkey	Eti Alüminyum	Seydizehir	37.84	20.24	15.27	6.15	9.43	2.23	[57]
USA	Alcoa	Arkansas	55.6	12.15	4.5	4.5	2–5	–	[57]
UK	Alcan	Lynemouth	46.0	20.0	5.0	6.0	8.0	1.0	[69]
Ukraine	Nikolaev	Halytsynove	39–43	17–19	9.5–11.1	4.4–5.6	6.2–6.9	7.6–9.5	[70]

## Data Availability

The data presented in this study are available on request from the corresponding author.
